# Clinical Significance of Circulating Tumor Cells in Patients with Esophageal Cancer

**DOI:** 10.14789/jmj.JMJ21-0049-OA

**Published:** 2022-08-01

**Authors:** HIROMI KITANO, MOTOMI NASU, TAKASHI HASHIMOTO, MASAHIKO TSURUMARU, YOSHIAKI KAJIYAMA

**Affiliations:** 1 Department of Esophageal and Gastroenterological Surgery, Juntendo University Graduate School of Medicine, Tokyo, Japan; 1 Department of Esophageal and Gastroenterological Surgery, Juntendo University Graduate School of Medicine, Tokyo, Japan

**Keywords:** esophageal cancer, hematogenous metastasis, circulating tumor cells, cellsearch^®^

## Abstract

**Objective:**

In recent years, circulating tumor cells (CTCs) have attracted attention for prediction of metastasis in breast, prostate, and colon cancers. This study aimed to investigate whether detection of CTCs could be prognostic factor in esophageal cancer.

**Methods:**

This study involved 38 patients treated at Juntendo University from May 2010 to April 2013 who provided consent. CTCs were measured using CellSearch^®^ system in preoperative peripheral blood. Clinicopathological parameters and prognostic factors were retrieved from our medical records.

**Results:**

CTCs were detected in 6 of 38 patients (15.8%). Among patients' characteristics and clinicopathological features, CTC-positive group had higher serum SCC levels and tended to have more advanced cStages than the CTC-negative group. The CTC-negative group showed better survival curves than CTCs positive-group in both overall survival (OS) and disease-free survival (DFS) although the differences were not statistically significant. CTCs positivity has a possibility to be prognostic marker according to multivariable analysis of OS and DFS.

**Conclusion:**

Although this study has some limitations, our results suggest that CTCs in preoperative peripheral blood has potential to be a prognostic marker for esophageal cancer.

## Introduction

Among various cancers, approximately 25,920 new esophageal cancer cases are reported in Japanese national surveillance of 2018^1)^, and this number is steadily increasing^2)^. Lymph node metastasis is the most common metastasis in resectable esophageal cancer; however, recurrence occurs in various sites. Of these, distant recurrences occur frequently and define prognosis after radical surgery with extensive lymph node dissection.

We sometimes encounter patients whose tumor marker levels in peripheral blood had increased before recurrence, or metastasis was detected by diagnostic imaging such as computed tomography, ultrasonography, magnetic resonance imaging, and positron emission tomography. Thus, more sensitive modalities are expected in clinical practice to detect the so-called “micrometastasis.” One of these micrometastases are circulating tumor cells (CTCs). In this decade, immunohistochemical methods and polymerase chain reaction methods have been used to verify the presence of CTCs in peripheral blood in advanced cancers; however, there is no established detection method^3)^.

Recent studies have shown CTCs in peripheral blood of metastatic patients might be suggest poor prognosis^4-6)^, and if CTC detection becomes possible in clinical practice, it will be useful for assessing a prognosticator and predicting early therapeutic effects. Although the clinical significance of CTC detection technology had not been established, CellSearch^®^ system (MENARINI, Itary) has good reproducibility and can detect even a single CTC in a 7.5-ml peripheral blood sample^7)^. In breast, colorectal, and prostate cancers, CTC counts before treatment and after the initial therapy correlate strongly with progression-free survival and overall survival (OS), moreover, therapeutic effects and prognosis can be predicted by measuring the CTCs count^4-9)^. As a result, the CellSearch^®^ system has been approved by the United States Food and Drug Administration (FDA) to test for CTCs in breast, prostate, and colorectal cancers. This study aimed to investigate whether detection of CTCs with the CellSearch^®^ system could similarly be useful to predict prognosis in esophageal cancer.

## Methods

### Patients

This study involved 38 esophageal cancer patients treated at the Juntendo University Hospital Department of Esophageal and Gastroenterological Surgery from May 2010 to April 2013. All patients had been pathologically diagnosed with esophageal cancer before treatment. The exclusion criteria are as follows: (1)multiple primaries (multicentric esophageal cancer are included); (2) non primary cases; (3) history of any cancer within 5years.

Written informed consents were obtained from all enrolled patients before this study. This study was approved by the Ethical Committee of Juntendo University Hospital (No.12-80).

Clinicopathological data were retrospectively retrieved from our database and electronic medical records. Tumor stage was assessed according to the International Union Against Cancer (UICC) TNM classification 7^th^ edition for esophageal cancer^10)^ from findings of gastrointestinal endoscopy, upper gastrointestinal series, computed tomography, and endoscopic ultrasound.

### Measurement of Circulating Tumor Cells

A CellSearch^®^ system epithelial cell kit was used to detect rare CTCs in whole blood by immunomagnetic separation. The kit contains a magnetic bead-based capture reagent and an immunofluorescent staining reagent, and the magnetic beads contain nanoparticles with magnetic cores surrounded by a polymer layer coated with an epithelial cell adhesion molecule (EPCAM) antibody to enrich CTCs. After enrichment and concentration using immunomagnetic separation, staining reagents are added to detect CTCs. Anti-CK-PE is specific for the intracellular protein cytokeratin (specific to epithelial cells), a nucleic acid stain (4′,6-diamidino-2-phenylindole [DAPI]) stains the cell nuclei, and anti-CD45 and APC react specifically with leukocytes.

The reagent and sample mixture are placed in a cartridge that sits in a MagNest^®^ holder that generates a magnetic field using the CellTracks AutoPrep system (MENARINI, Itary). Magnetically labeled epithelial cells move to the surface of the cartridge due to the strong magnetic field generated by the MagNest^®^, the fluorescent images are captured by the Cell Tracks^®^ Analyzer II, and candidates stained with both CK-PE and DAPI inside the cartridge are displayed. Images are presented in a gallery format for the final cell classification. The images are classified as tumor cells based on morphology and phenotype (EPCAM+, CK+, DAPI+, and CD45−).

A 20-ml blood sample was collected before all treatment, and 10 ml was distributed among two Cell Save storage tubes. Subsequently, 7.5 ml of the 10 ml blood sample was transferred to a conical test tube, 6.5 ml of diluent was added, and the conical test tube was capped and mixed by inverting five times. The sample was centrifuged at 800 rpm for 10 min with the centrifuge brake released. The sample was placed in the CellTracks AutoPrep device of the CellSearch^®^ system for processing within 1 hour.

The CellTracks^®^ AutoPrep system specifically isolates and extracts epithelial cells from the many cells in the blood using magnetic particles that consist of antibodies for EPCAM bound to iron nanoparticles. The isolated epithelial cells are bound by a fluorescently labeled cytokeratin monoclonal antibody, and the nuclei are stained with DAPI fluorescent DNA stain. Similarly, leukocytes are bound by fluorescently labeled CD45 antibodies to distinguish them from CTCs. The CTC reaction solution is transferred into a cartridge that sits in a device with a fixed magnet called the MagNest and then placed in the CellTracks Analyzer II^®^ of the CSS to analyze the test results. The magnetic force generated by the magnet in the MagNest moves the CTCs captured by the ferrofluid to the top of the cartridge. The data of the fluorescent colors that appear on the upper surface of the cartridge are processed into fluorescence images for analysis and assessment. We defined CTC positive when CTC count is at least one in 7.5 ml of blood sample.

We measured serum tumor makers (cytokeratin fragment [CYFRA], squamous cell carcinoma antigen [SCC], carbohydrate antigen 19-9 [CA19-9], and carcinoembryonic antigen [CEA]) of same samples. Blood samples for CTCs and tumor makers were collected from first visit of our department to beginning of any treatment.

### Statistical Analyses

All statistical analyses were performed using IBM SPSS advanced statistics ver.25. The chi- square test was applied for the differences of patients’ characteristics and CTCs status between the CTCs-positive and the CTCs-negative groups. Serum CYFRA, CA19-9, SCC and CEA levels are divided into two groups of over and within normal range. Differences were considered significant at p-value <0.05. Values are expressed as median (range).

OS and disease-free survival (DFS) rates were calculated using the Kaplan–Meier method, and univariate analyses were performed by the Log-rank test. The DFS was defined as duration (days) from the date of surgery (or the first treatment day of chemotherapy or chemoradiotherapy patients) to the first relapse of cancer or death from any cause. Cox hazards regression analysis was performed to evaluate the effect of CTCs positivity and other parameters to OS and DFS. In Cox hazards regression analysis, cTMN stages are divided into the cStageI +II and III+IV groups.

## Results

The demographics of the patients in the entire cohort are shown in Table 1. Surgical treatments were performed in 29 patients (76.3%), but cohort also included patients undergoing endoscopic treatment, chemotherapy and chemoradiotherapy. Eight of 29 patients who underwent esophagectomy received neoadjuvant therapy.

**Table 1:** Patients' characteristics

Number of cases	38 cases	
Age	66 (47-84) years old	
Sex	Male	33 (86.8%)
	Female	5 (13.2%)
Treatment	Surgery	35
	Esophagectomy with three(two) -fields lymph node dissection	28 (73.7%)
	Laryngectomy with cervical esophagecto-my	1 (2.6%)
	Endoscopic treatment	6 (15.8%)
	Chemotherapy alone/ Chemoradiotherapy	3 (7.8%)
Primary tumor location	Ce	4 (10.5%)
	Ut	7 (18.4%)
	Mt	14 (36.8%)
	Lt	13 (34.2%)
Predominant Histological type	Well-differentiated SqCC	10 (26.3%)
	Moderately differentiated SqCC	21 (55.3%)
	Poorly differentiated SqCC	2 (5.3%)
	Others (adenocarcinoma, Basaloid carcinoma)	5 (13.2%)
cT classification*	cT1	15 (39.5%)
	cT2	4 (0.5%)
	cT3	15 (39.5%)
	cT4	4 (10.5%)
Clinical stage *	Ⅰ	15 (39.5%)
	II	5 (13.2%)
	III	14 (36.8%)
	IV	4 (10.5%)

* UICC TNM Classification of Malignant Tumors, 7^th^ ed Ce: Cervical esophagus, Ut: Upper thoracic esophagus, Mt: Middle thoracic esophagus, Lt: Lower thoracic esophagus SqCC: Squamous cell carcinoma

Among these 38 patients, CTCs were detected in only 6 (15.7%) patients, the CTC counts were 1/ 7.5ml of blood sample in 4 patients, 2 in one patient, and 190 in one patient. Therefore, we divided the patients into the CTC-positive and CTC-negative groups. (Table 2). CTC-positive group had higher serum SCC level than the CTC-negative group (p= 0.014), and also CTCs-positive group tended to have more advanced cStage than CTCs-negative group (p=0.055). There were no significant differences between these two groups in other clinicopathological factors.

**Table 2:** Comparison of CTC-positive and CTC-negative cases

Clinicopathological factors	Variables	CTC-negative	CTC-positive	p-Value
Sex	Male / Female	28/ 4	5/ 1	0.788
Age		66.0	67.0	0.110
Main treatment	Operation/ ESD/ CRT/ other	26/ 4/ 2/ 0	2/ 2/ 1/ 1	0.630
Primary tumor location	Ce/ Ut/ Mt/ Lt	3 /6/ 11/ 12	1/ 1/ 3/ 1	0.727
Predominant Histological type	Well/ Mod/ Poor/ other	9/ 18/ 2/ 3	1/ 4 /0/ 1	0.732
cT classification*	T1/ T2/ T3/ T4	13/ 4/ 13/ 2	2/ 0/ 2/ 2	0.257
cN classification*	N0/ N1/ N2/ N3	13/ 7/ 10/ 2	2/ 0/ 2/ 2	0.171
cStage*	I/ II/ III/ IV	12/ 4/ 14/ 2	2/ 0/ 1/ 3	0.055
CYFRA	high/ normal range	3/ 28**	2/ 4	0.325
CA19-9	high/ normal range	2/ 30	1/ 5	0.385
SCC	high/ normal range	3/ 28**	3/ 3	0.014
CEA	high/ normal range	10/ 20**	0/ 6	0.096

ESD: Endoscopic submucosal resection, CRT: Chemoradiotherapy Ce: Cervical esophagus, Ut: Upper thoracic esophagus, Mt: Middle thoracic esophagus, Lt: Lower thoracic esophagus Well: Well differentiated Squamous cell carcinoma, Mod: Moderately differentiated Squamous cell carcinoma, poor: poorly differentiated Squamous cell carcinoma CYFRA: cytokeratin fragment, CA19-9: carbohydrate antigen 19-9, SCC: squamous cell carcinoma antigen, CEA: and carcinoembryonic antigen * UICC TNM Classification of Malignant Tumors, 7^th^ ed **not measured in some cases

**Figure 1:**
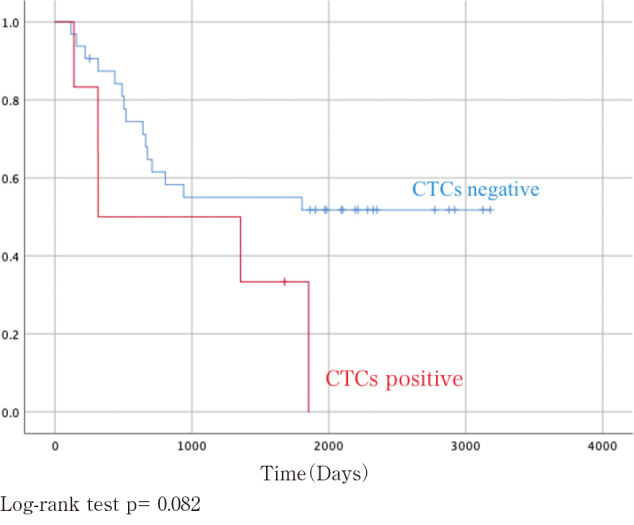
Overall survival (OS) in the CTC-negative and -positive groups

**Figure 2:**
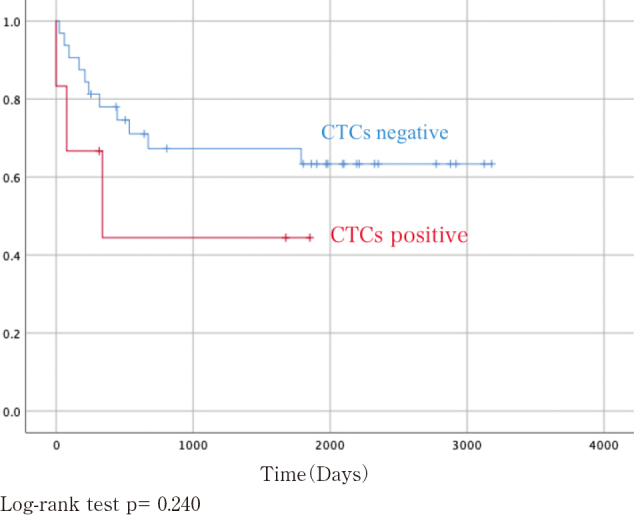
Disease-free survival (DFS) in the CTC-negative and -positive groups

Regarding survivals, the CTCs-negative group showed better survival curves than CTCs positive- group in both OS and DFS, however the differences were not statistically significant (Figure 1 and 2). In multivariate analysis, we chosen CTCs status, cStage, serum SCC level and CEA level as independent variables based on p-value of less than 0.2. Cox hazards regression model showed that CTCs status was likely to be a prognostic factor, but not statistically significant (OS: Hazard Ratio (HR) =0.358, 95% confidence interval (CI) 0.122-1.502, p =0.062, DFS: HR=0.358, 95% (CI) 0.152-2.323, p =0.455), as shown in Table 3A and 3B.

**Table 3:** Long-term outcome and CTCs

A) Overall survival					
Clinicopathological factors	Variables	p-Value	Exp(b)	95% CI Lower	95% CI Upper
CTCs	Positive/ negative	0.062	0.358	0.122	1.502
cStage*	I+II/ III+IV	0.006	2.237	1.259	3.973
CEA	(continuous)	0.519	1.016	0.968	1.067
SCC	(continuous)	0.647	1.006	1.259	3.973

B) Disease-free survival					
Clinicopathological factors	Variables	p-Value	Exp(b)	95% CI Lower	95% CI Upper
CTCs	Positive/ negative	0.455	0.594	0.152	2.323
cStage*	I+II/ III+IV	0.021	11.725	1.458	94.266
CEA	(continuous)	0.325	1.026	0.975	1.079
SCC	(continuous)	0.802	1.003	0.978	1.029

SCC: squamous cell carcinoma antigen, CEA: carcinoembryonic antigen * UICC TNM Classification of Malignant Tumors, 7^th^ ed

## Discussion

In this study, we investigated the relationships between CTCs and survivals. We could show that patients without CTCs had better survival than those of CTCs positive, however the difference was not statistically significant. In multivariate analysis regarding OS and DFS, we were able to demonstrate that CTCs positivity has a possibility to be prognostic marker. We speculated that these discrepancies might be from small sample size and the heterogeneity of patients’ background. Actually, our enrolled patients included those with early disease that can be treated by endoscopy and those with distant metastasis. We assume that the differences in survivals between CTCs positive and CTCs negative groups would be clear if patients’ backgrounds were limited to some extent.

In addition, we investigated the relationships between the detection of CTCs using the CellSearch^®^ system and clinicopathological factor for patients with esophageal cancer. Although the CellSearch^®^ system is approved by the FDA for some adenocarcinomas (breast, colorectal, and prostate cancers)^4-9)^, little has been reported on SqCC. The CTCs detection rate in esophageal squamous cell carcinoma is so low that few studies have been conducted in this area^11-13)^. Actually, the CTCs positivity rate in the present study of esophageal cancer was low, 15.7%. This could be due to the distribution of the patients’ characteristics with relatively early-stage cancers. Among this cohort, 39.5% had a depth of T1, 39.4% had no lymph node metastasis, and 31.5% had cStage I cancer.

According to our results, CTCs status was significantly related to the serum SCC level. SCC is widely found in epithelial cells and known as tumor maker of squamous cell carcinoma, not only esophagus but also lung, head and neck and others, known to associated with metastasis and poor prognosis^14-16)^. In addition, in some previous studies, SCC mRNA in lymph nodes or peripheral blood were used as a marker of micrometastasis of squamous cell carcinoma. Thus, CTCs counts, as micrometastasis, might be related with SCC level.

This study has some limitations. First, only a small number of patients were enrolled this study. Second, this study utilized a retrospective design. Recently, the sensitivity of CTCs detection reported to improve in other cancers^17, 18)^, therefore, we would consider large sample sizes to the analysis in future.

In conclusion, our results suggest that CTCs in preoperative peripheral blood has possibility to prognostic marker of esophageal cancer. Further studies are needed to confirm this finding.

## Funding

The author(s) received no financial support for the research.

## Author contributions

HK corrected blood samples, interpreted the patient data, and was a major contributor in writing the manuscript. MT and YK recruited patients. The article was revised by MN and the final manuscript has been approved by all authors.

## Conflicts of interest statement

The authors declare that they are no conflicts of interest.
